# Plasma and intrapulmonary pharmacokinetics of cefepime and taniborbactam in healthy adult participants

**DOI:** 10.1128/aac.00493-25

**Published:** 2025-06-06

**Authors:** Keith A. Rodvold, Mark H. Gotfried, Philip Sabato, Tim Henkel, Paul C. McGovern

**Affiliations:** 1 University of Illinois Chicago 14681, Chicago, Illinois, USA; 2 Pulmonary Associates 92437, Phoenix, Arizona, USA; 3 Venatorx Pharmaceuticals 540451, Malvern, Pennsylvania, USA; Providence Portland Medical Center, Portland, Oregon, USA

**Keywords:** cefepime, taniborbactam, pharmacokinetics, epithelial lining fluid

## Abstract

Cefepime-taniborbactam is being developed for the treatment of serious multidrug-resistant (MDR) Gram-negative bacterial infections. This study determined and compared plasma and epithelial lining fluid (ELF) concentrations of cefepime and taniborbactam in 30 healthy adult participants. The dosing regimen was 2 g cefepime/0.5 g taniborbactam administered as a 4 h intravenous infusion every 8 h for a total of six doses. Mean plasma and ELF concentration values with the four aspirates at each bronchoalveolar lavage (BAL) sampling time (1, 3, 4.25, 5, 6, and 8 h) were used to estimate the area under the concentration-time curve (AUC_0–8_). The mean AUC_0–8_ values of unbound plasma concentrations of cefepime and taniborbactam were 262.2 and 84.77 µg·h/mL, respectively. The drug penetration ratios of ELF to unbound plasma concentrations (DPR_ELF/plasma_) were based on the AUC_0–8_ values of six different BAL aspirate calculations: single aspirates (for the first, second, third, and fourth BAL sample) and pooled aspirates (BAL samples 2+3+4 and 1+2+3+4). The AUC_0–8_ values for ELF for individual and pooled aspirates ranged from 51.62 to 97.86 µg·h/mL for cefepime and 13.14 to 21.69 µg·h/mL for taniborbactam. The DPR_ELF/plasma_ ranged from 0.197 to 0.373 for cefepime and 0.153 to 0.253 for taniborbactam and was dependent on which aspirate of recovered BAL fluid was used. The highest and lowest values of AUC_0–8_ values for ELF and DPR_ELF/plasma_ for cefepime and taniborbactam were observed with aspirate 1 and aspirate 4, respectively. These results support further consideration of cefepime-taniborbactam as a potential treatment for bacterial pneumonia caused by susceptible MDR Gram-negative pathogens.

**CLINICAL TRIALS:**

This study is registered with ClinicalTrials.gov as NCT04951505.

## INTRODUCTION

Taniborbactam (formerly VNRX-5133) is a broad-spectrum bicyclic boronate β-lactamase inhibitor with potent inhibitory activity against Ambler classes A, B, C, and D β-lactamases (1). Taniborbactam, in combination with the fourth-generation cephalosporin cefepime, is expected to address the serious unmet medical need for a safe and effective therapy for the treatment of infectious diseases due to multidrug-resistant (MDR) Gram-negative bacteria. Cefepime-taniborbactam has potent *in vitro* activity, including extended-spectrum β-lactamase-producing organisms and carbapenem-resistant *Enterobacterales* and *Pseudomonas aeruginosa* (2–5). Cefepime-taniborbactam is being developed for the treatment of serious Gram-negative infections caused by MDR Gram-negative bacteria in complicated urinary tract infections (cUTIs) and may be used for the treatment of hospital-acquired bacterial pneumonia/ventilator-associated bacterial pneumonia. Cefepime-taniborbactam has demonstrated composite microbiologic and clinical success in a phase 3 clinical trial (CERTAIN-1) for the treatment of patients with cUTIs (6).

Epithelial lining fluid (ELF) has been advocated as an important infection site for common extracellular pathogens causing lower respiratory tract infections (7–10). Studies with bronchoscopy and bronchoalveolar lavage (BAL) have been performed to estimate the intrapulmonary penetration of antibiotics into the ELF. The primary objective of this study was to evaluate the steady-state plasma and ELF concentrations of cefepime and taniborbactam in healthy adult participants after six consecutive doses of cefepime-taniborbactam (2 g cefepime/0.5 g taniborbactam) administered as 4 h intravenous infusions every 8 h. The analyses included a comparison of intrapulmonary ELF concentrations when single and pooled BAL fluid aspirates were used. The secondary objective of this study was to assess the safety and tolerability of cefepime and taniborbactam in healthy adult participants.

## RESULTS

### Study population

Thirty healthy adult participants comprised the safety and pharmacokinetic populations. The age of 21 male and 9 female participants ranged from 27 to 55 years (mean ± standard deviation [SD]: 40 ± 8 years). Participants had a mean (SD) weight and body mass index of 81.8 (12.8) kg and 27.5 (3.2) kg/m^2^, respectively. The estimated creatinine clearance determined by the Cockcroft-Gault equation ranged from 72 to 161 mL/min (mean ± SD: 114 ± 20 mL/min) (11). Demographic data were generally similar across BAL sampling cohorts (Table S1).

### Safety

Four-hour intravenous infusions of 2 g cefepime in combination with 0.5 g taniborbactam every 8 h for six consecutive doses were well tolerated in healthy adult participants. Three participants (10%) experienced a total of three treatment-emergent adverse events (TEAEs) over the course of the study, of which two TEAEs (dry mouth and blood pressure increased) were possibly related to the study treatment and one TEAE (infusion site pain) was considered not related to the administration of study drug. All TEAEs were mild in severity. There were no serious adverse events or deaths reported in the study. The mean changes from baseline in hematology, biochemistry, coagulation, and urinalysis values, electrocardiogram (ECG) parameters, vital signs, and physical examination findings were not notably different during the study.

### Plasma pharmacokinetics

**Fig 1:**
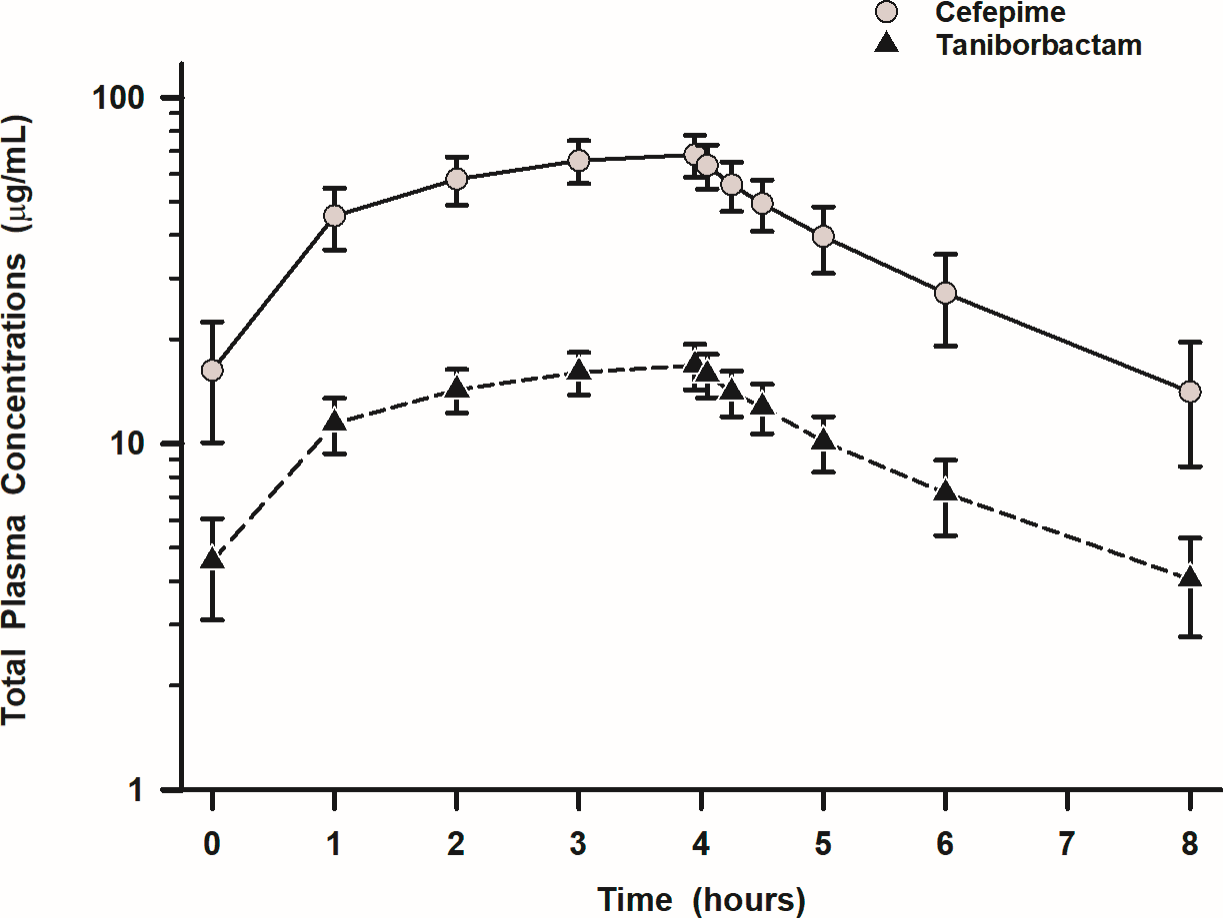
Total plasma concentration profile (arithmetic mean ± SD) for cefepime (gray circle) and taniborbactam (black triangles) after the sixth 4 h intravenous infusion of 2 g cefepime in combination with 0.5 g taniborbactam every 8 h. The *y*-axis is a semi-log scale.

Mean (±SD) total plasma concentrations for cefepime and taniborbactam before, during, and after the sixth 4 h intravenous infusion of 2 g cefepime in combination with 0.5 g taniborbactam every 8 h are exhibited in Fig. 1. Plasma concentrations prior to the sixth dose and at 8 h following that dose were similar and indicated that steady state had been achieved. All participants had measurable plasma cefepime and taniborbactam trough concentrations before the third, fourth, and fifth doses (data not shown) and were similar in value to those observed with the sixth dose. The mean (±SD) plasma pharmacokinetic parameters of cefepime and taniborbactam for total plasma concentration-time profiles are presented in Table 1.

**TABLE 1:** Non-compartmental pharmacokinetic parameters of cefepime and taniborbactam in plasma after the sixth 4 h intravenous infusion of 2 g cefepime in combination with 0.5 g taniborbactam every 8 h^*a*^

	C_max_ (µg/mL)	T_max_ (h)	C_min_ (µg/mL)	AUC_0–8_ (µg·h/mL)	CL (L/h)	V_ss_ (L)	t_1/2_ (h)
Cefepime^*b*^	69.18 ± 9.73	3.95 (3.00, 4.00)	14.10 ± 5.52	334.3 ± 58.3	6.14 ± 1.00	14.82 ± 2.84	1.90 ± 0.36
Taniborbactam^*b*^	17.04 ± 2.46	3.95 (3.00, 4.07)	4.05 ± 1.29	84.77 ± 13.58	6.03 ± 0.90	16.31 ± 3.28	2.10 ± 0.37

^*a*^ Data are expressed as arithmetic mean ± SD except for T_max_ (median [range]).

^*b*^ 30 parameter values for each listing.

### Urea concentrations and apparent ELF volumes

Table 2 summarizes the mean (±SD) measurements of urea concentrations in plasma and BAL, volume of aspirated BAL fluid, apparent ELF volumes, and duration of time for the collection of each aspirate. Higher urea concentrations in BAL fluid and larger apparent ELF volumes were observed with aspirates 2, 3, and 4 compared to aspirate 1. Aspirates 3 and 4 were also associated with longer durations of time for the collection of BAL aspirates and greater recovered BAL volume.

**TABLE 2:** Urea concentrations in plasma and BAL, volume of BAL aspirates, and calculated ELF volume (*N* = 30)^*a*^

Measurement	Aspirate 1	Aspirate 2	Aspirate 3	Aspirate 4
Plasma urea (µg/mL)	316.12 ± 67.55	316.12 ± 67.55	316.12 ± 67.55	316.12 ± 67.55
BAL urea (µg/mL)	4.42 ± 2.36	7.37 ± 4.59	8.69 ± 5.01	10.30 ± 5.98
BAL volume (mL)	20.9 ± 5.6	34.8 ± 7.1	38.7 ± 5.9	38.5 ± 7.0
Duration (min)^*b*^	2.1 ± 0.6	1.2 ± 0.4	3.6 ± 0.8	4.2 ± 1.3
Apparent ELF volume (mL)	0.30 ± 0.18	0.80 ± 0.45	1.01 ± 0.48	1.18 ± 0.56

^*a*^ Data are expressed as arithmetic mean ± SD.

^*b*^ Approximate duration of time to collect aspirate following saline installation.

### ELF concentrations and penetration ratios

**Fig 2:**
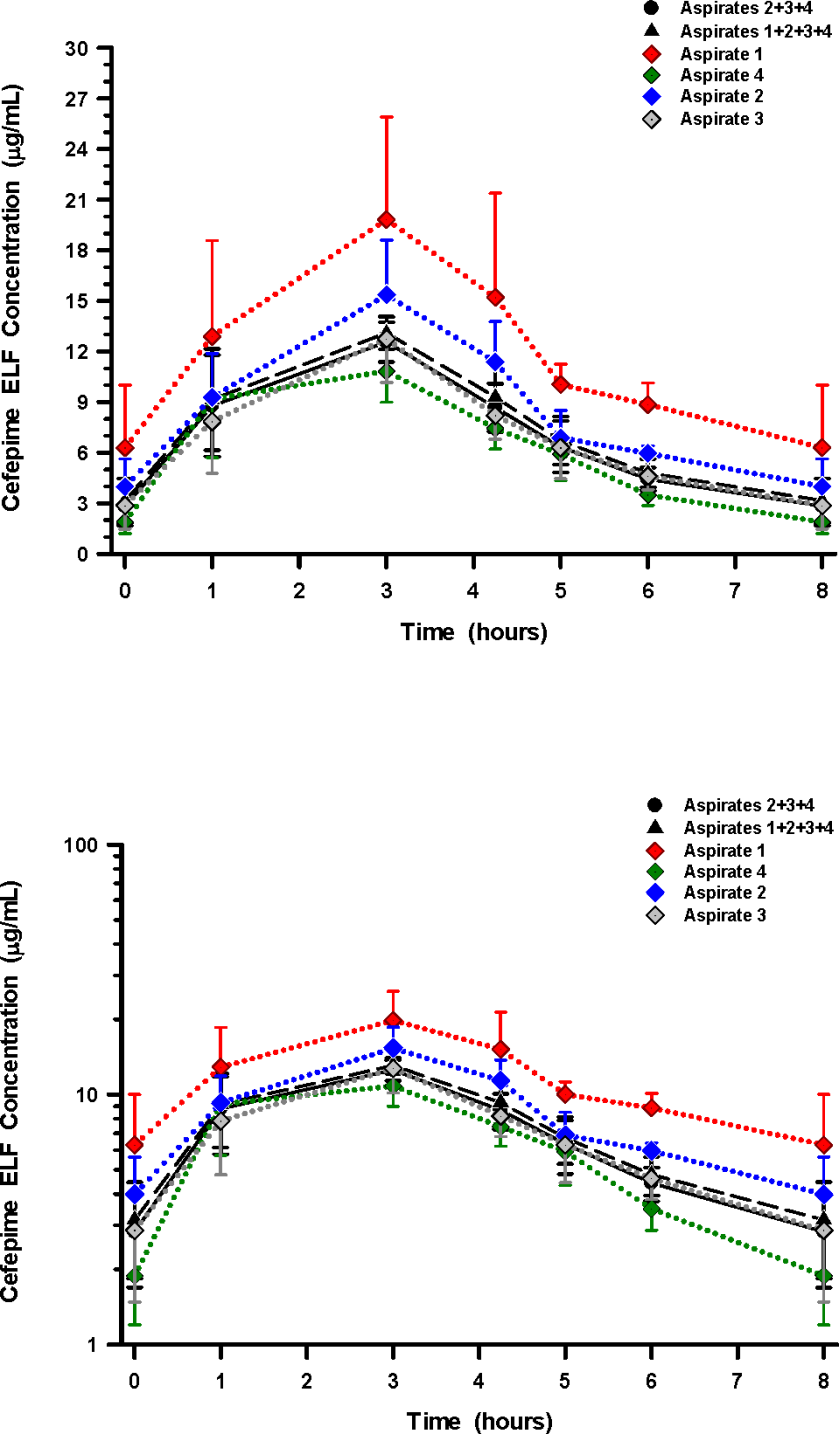
Concentrations (arithmetic mean ± SD) of estimated cefepime in ELF for individual and pooled aspirates at the scheduled BAL sampling times after the start of the sixth 4 h intravenous infusion of 2 g cefepime in combination with 0.5 g taniborbactam every 8 h. The *y*-axis of the top figure is a linear scale, and the bottom figure is a semi-log scale.

**Fig 3:**
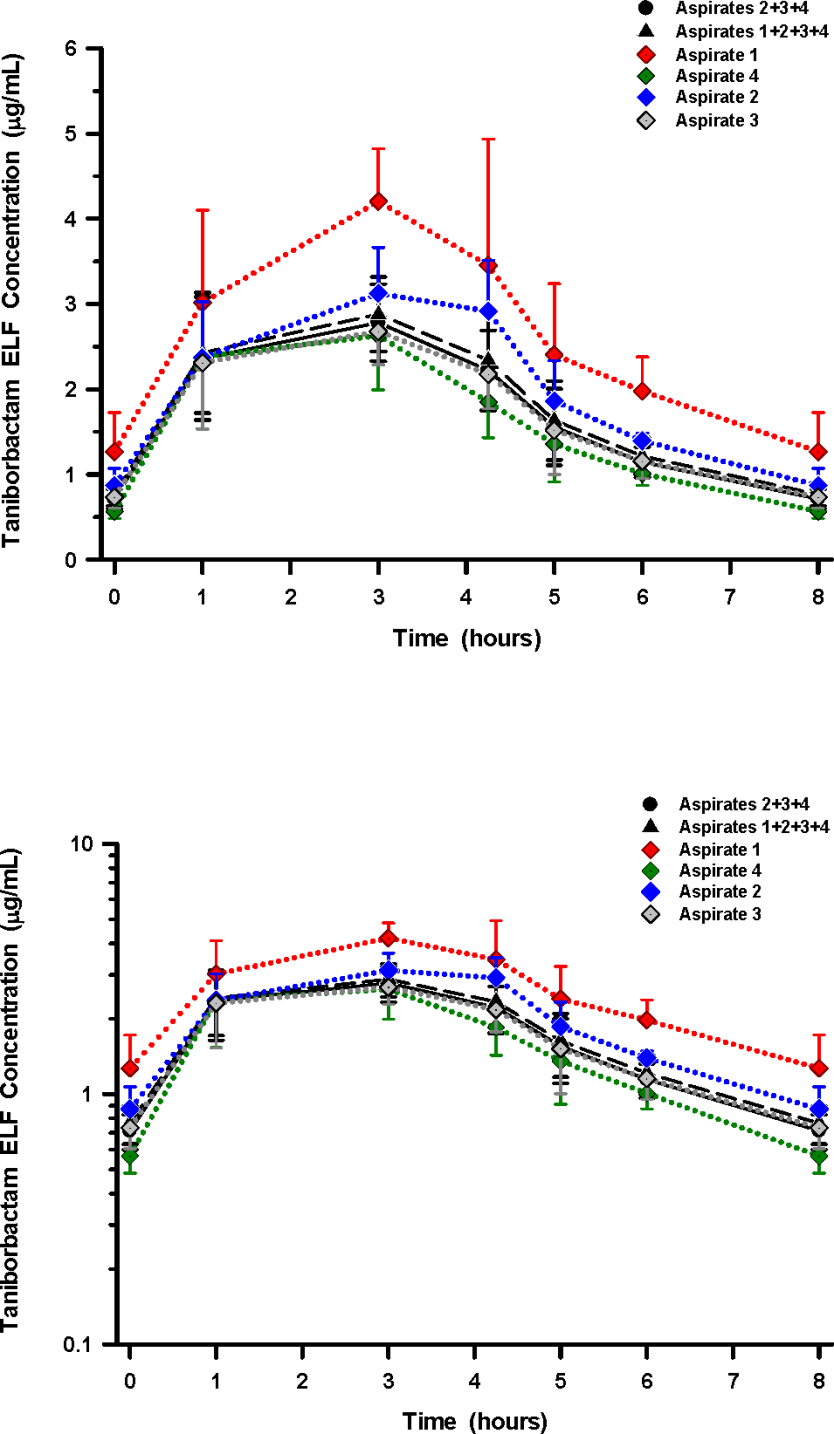
Concentrations (arithmetic mean ± SD) of estimated taniborbactam in ELF for individual and pooled aspirates at the scheduled BAL sampling times after the start of the sixth 4 h intravenous infusion of 2 g cefepime in combination with 0.5 g taniborbactam every 8 h. The *y*-axis of the top figure is a linear scale, and the bottom figure is a semi-log scale.

Mean (±SD) concentrations of cefepime and taniborbactam in ELF for individual and pooled aspirates throughout the bronchopulmonary sampling times are displayed in Fig. 2 and 3, respectively. The highest and lowest ELF concentrations were consistently observed with aspirates 1 and 4, respectively. Tables S2 and S3 summarize the mean (±SD) concentrations of cefepime and taniborbactam for BAL aspirates. The area under the concentration-time curve (AUC_0–8_) values for ELF for individual and pooled aspirates ranged from 51.62 to 97.86 µg·h/mL for cefepime (Table 3) and 13.14 to 21.69 µg·h/mL for taniborbactam (Table 4). The drug penetration ratio (DPR) of ELF to unbound plasma concentrations based on AUC_0–8_ (DPR_ELF/plasma_) for the six BAL aspirate calculations ranged from 0.197 to 0.373 for cefepime (Table 3) and 0.153 to 0.253 for taniborbactam (Table 4), respectively. The highest and lowest values of AUC_0–8_ values for ELF and DPR_ELF/plasma_ for cefepime and taniborbactam were observed with aspirate 1 and aspirate 4, respectively. The mean (±SD) ratios of ELF to the simultaneous unbound plasma concentrations of cefepime and taniborbactam for BAL aspirates and at assigned BAL sampling times are reported in Tables S4 and S5, respectively.

**TABLE 3:** Exposure and drug penetration ratio for cefepime in plasma and ELF for BAL aspirates^*a*^^,^^*d*^

Parameter	Aspirate 1	Aspirate 2	Aspirate 3	Aspirate 4	Aspirates 2+3+4	Aspirates 1+2+3+4
AUC_0–8_ plasma^*a*^	337.9	337.9	337.9	337.9	337.9	337.9
*f*AUC_0–8_ plasma^*a*^^,*c*^	262.2	262.2	262.2	262.2	262.2	262.2
AUC_0–8_ ELF^*a*^	97.86	70.81	56.99	51.62	58.31	61.73
DPR_ELF/plasma_^*b*^	0.373	0.270	0.217	0.197	0.222	0.235
t_1/2-ELF_ (h)	3.24	2.74	2.50	1.85	2.36	2.47

^*a*^ Mean total concentration value at each BAL sampling time were determined and data from all sampling times were combined into a single data set to calculate the AUC_0–8_ value of each matrix.

^*b*^ DPR into ELF (ratio of AUC_0–8_ of ELF to *f*AUC_0–8_ unbound plasma of cefepime).

^*c*^ Value used for the unbound fraction (f) of cefepime in plasma was 0.776.

^*d*^ Units for AUC_0–8_ and *f*AUC_0–8_ as µg·h/mL.

**TABLE 4:** Exposure and drug penetration ratio for taniborbactam in plasma and ELF for BAL aspirates^*a*^^,^^*d*^

Parameter	Aspirate 1	Aspirate 2	Aspirate 3	Aspirate 4	Aspirates 2+3+4	Aspirates 1+2+3+4
AUC_0–8_ plasma^*a*^	85.73	85.73	85.73	85.73	85.73	85.73
*f*AUC_0–8_ plasma^*a*^^,*c*^	85.73	85.73	85.73	85.73	85.73	85.73
AUC_0–8_ ELF^*a*^	21.69	16.49	14.09	13.14	14.35	14.99
DPR_ELF/plasma_^*b*^	0.253	0.192	0.164	0.153	0.167	0.175
t_1/2-ELF_ (h)	2.74	2.27	2.49	2.24	2.37	2.41

^*a*^ Mean total concentration value at each BAL sampling time was determined, and data from all sampling times were combined into a single data set to calculate the AUC_0–8_ value of each matrix.

^*b*^ DDPR into ELF (ratio of AUC_0–8_ of ELF to *f*AUC_0–8_ unbound plasma of taniborbactam).

^*c*^ Value used for the unbound fraction (f) of taniborbactam in plasma was 1.0.

^*d*^ Units for AUC_0–8_ and *f*AUC_0–8_, is expressed in µg·h/mL.

## DISCUSSION

The plasma pharmacokinetic parameters for single and multiple doses of the combination of 2 g of cefepime and 0.5 g of taniborbactam in healthy adult participants have recently been reported (12, 13). Following the administration of this combination as 2 h intravenous infusions, similar values for AUC_0–8_, CL, V, and elimination half-life (t_1/2_) of cefepime were observed in our study (Table 1). Lower values of C_max_ and higher values of C_min_ were observed in this study and reflect the use of a 4 h intravenous infusion for cefepime administration.

The mean (±SD) plasma pharmacokinetic parameter values of taniborbactam observed by Asempa et al. were comparable to our study (13). The difference between the 2 h versus 4 h intravenous infusion of taniborbactam resulted in lower values of C_max_ and higher values of C_min_. Concentrations of cefepime and taniborbactam in ELF were lower than total or unbound plasma concentrations (unbound fraction values were 0.776 and 1.00 for cefepime and taniborbactam, respectively) (13). Asempa et al. reported DPR_ELF/plasma_ for cefepime and taniborbactam as 0.30 and 0.17, respectively (13). These investigators followed the traditional handling of BAL sample collection, where the first BAL aspirate was discarded (in order to more accurately measure alveolar macrophage concentrations due to epithelial cell contamination), and the subsequent three BAL aspirates were pooled and processed together for measuring antibiotic and urea concentrations in BAL fluid. The respective observed DPR_ELF/plasma_ of cefepime and taniborbactam in our study was 0.22 and 0.17 when a similar approach (ie, BAL aspirates 2+3+4) was used for the calculations. Higher DPR_ELF/plasma_ (0.61 and 0.39) of cefepime has been observed when 2 h intravenous infusions were administered to healthy adult participants and combined with the beta-lactamase inhibitors enmetazobactam and zidebactam, respectively (14, 15). The observed differences in DPR_ELF/plasma_ estimates in these two studies were likely explained by a combination of many factors including variations in duration of infusion (i.e., 2 versus 4 h), differences in dwell times, number of BAL samples (i.e., 4 versus 6 and 7 samples) available for pharmacokinetic analysis, and limitations of the urea dilution methodology.

Alveolar macrophage concentrations of cefepime and taniborbactam were not determined in this study and have been previously reported (13). The aims of this study included the determination of intrapulmonary pharmacokinetics of a 4 h intravenous infusion of cefepime-taniborbactam (2 g cefepime and 0.5 g taniborbactam every 8 h) and assessment of single and pooled BAL fluid aspirates to determine ELF concentrations. The results permitted comparison of ELF concentrations and DPR_ELF/plasma_ estimates of cefepime and taniborbactam observed in previously reported human and animal studies (13–16).

Epithelial lining fluid concentrations of cefepime and taniborbactam in this study were determined for each BAL aspirate and by combining three and four BAL aspirates. The DPR_ELF/plasma_ ranged from 0.197 to 0.373 for cefepime and 0.153 to 0.253 for taniborbactam (Tables 3 and 4). The highest exposures in ELF were observed when only the first BAL aspirate was used to calculate AUC_0–8_, DPR_ELF/plasma_, or ratios of ELF to simultaneous unbound plasma concentration of cefepime and taniborbactam (Tables 3 and 4; Tables S4 and S5). The AUC_0–8_ values of ELF for the first BAL aspirate were 97.86 µg·h/mL and 21.69 µg·h/mL for cefepime and taniborbactam, respectively (Tables 3 and 4). In comparison, the AUC_0–8_ values of ELF for the combined BAL aspirates 2, 3, and 4 were 58.31 µg·h/mL and 14.35 µg·h/mL for cefepime and taniborbactam, respectively. These differences were explained by a large relative increase in urea ELF concentrations and apparent ELF volume in successive BAL aspirates 2, 3, and 4 compared to the first BAL aspirate.

A similar observation of higher ELF drug concentrations and lower ELF urea concentrations with the first BAL aspirate has been described with cefepime using a dog lung model (16). Bayat et al. indicated that ELF concentrations determined by the urea dilution method were lower and more variable than those obtained using the technetium-99m diethylenetriaminepenta-acetic acid (^99m^Tc-DTPA) method, particularly in dogs with normal, non-injured lungs. This study also suggested that normal, non-injured lungs may underestimate ELF concentrations, independent of the method, to estimate ELF volume.

Measurement of urea in BAL fluid and plasma has been commonly used for the correction of ELF dilution and determination of the apparent volume of ELF (7–10, 17). A major limitation of using urea is the overestimation of ELF volume and underestimation of drug or solute concentrations (8–10, 17). Compared to aspirate 1, concentrations of urea in BAL and the calculated apparent ELF volumes were higher in aspirates 2, 3, and 4 (Table 2). Our study complements other investigations suggesting that urea diffuses into the alveolar space during subsequent installations of BAL fluid (18–20). Haeger et al. suggested that the increase of urea seen in subsequent aliquots of BAL fluid from healthy adult participants may be due to incomplete mixing between BAL and ELF, resulting in more ELF being sampled with later aliquots (21). In addition, several other components of the lavage procedure can influence the diffusion of urea from plasma, including the duration of dwell time, amount of fluid instilled with each BAL, and percentage of instilled BAL returned (17). Aspirates 3 and 4 in our study were also associated with longer durations of dwell time and larger volumes of recovered BAL fluid. Whether or not the urea concentration in the first aspirate of recovered BAL fluid is the most accurate estimate of the volume of ELF and ELF antibiotic concentrations remains unknown. It is likely that reported antibiotic concentrations in healthy participants are conservative estimates of the actual amount of drug present in ELF since most intrapulmonary penetration studies of antibiotics have used pooled BAL fluid from aspirates 2, 3, and 4. Reported ELF concentrations from intrapulmonary penetration studies need careful consideration regarding the timing, volume, and number of aspirates collected, as well as the variability and limitations of the urea dilution method.

The *in vitro* susceptibilities of cefepime-taniborbactam against isolates of Enterobacterales (*n* = 13,731) and *Pseudomonas aeruginosa* (*n* = 4,619) have exhibited MIC_50_ and MIC_90_ values of 0.06 and 0.25 µg/mL (99.7% of isolates inhibited at ≤16 µg/mL) and 2 and 8 µg/mL (97.4% of isolates inhibited at ≤16 µg/mL), respectively (4). The addition of a fixed 4 µg/mL concentration of taniborbactam to cefepime reduced the MIC_90_ value from 16 to 0.25 µg/mL (64-fold reduction) for Enterobacterales isolates and from 32 to 8 µg/mL (fourfold reduction) for *Pseudomonas aeruginosa* isolates. Pharmacokinetic-pharmacodynamic target parameters correlated to antibacterial activity include the ratio of the 24 h AUC (AUC_0–24_) of unbound (*f*) taniborbactam to the MIC value (*f*AUC_0–24_/MIC) and percent time (%T) of unbound cefepime concentration above the MIC value (%*f*T > MIC) (13, 22–25). When cefepime-taniborbactam was administered in a murine neutropenic infection model of the lung to reflect human concentration-time profiles, the respective median plasma *f*AUC_0–24_/MIC ratios of taniborbactam needed for bacterial stasis and 1−log_10_ CFU reduction from baseline were 0.96 and 4.03 for Enterobacterales and 1.35 and 3.02 for *Pseudomonas aeruginosa* (22). The ranges of DPR_ELF/plasma_ for taniborbactam and cefepime in this murine infection model were 0.85 to 1.11 and 0.61 to 0.64, respectively. Similar median plasma *f*AUC_0–24_/MIC values of taniborbactam were reported in a murine neutropenic infection model of the thigh for Enterobacterales (1.18 and 2.62, respectively); however, the values for *Pseudomonas aeruginosa* were considerably lower (0.29 and 0.46, respectively) (25). These *f*AUC_0–24_/MIC ratios of taniborbactam based on plasma target values are likely to be achieved with the observed concentration-time proﬁles in our study of healthy adult participants. Preclinical ELF pharmacokinetic-pharmacodynamic targets and assessment of the probability of target attainment are still needed.

The combination of cefepime 2 g and taniborbactam 0.5 g administered as a 4 h infusion every 8 h was well tolerated by 30 healthy participants in this pharmacokinetic study. A total of three participants experienced three TEAEs, including dry mouth, blood pressure increase, and pain at the infusion site. All observed TEAEs were mild in severity, and two TEAEs were considered possibly related to study drugs. The most common TEAEs reported in a large, phase 3 clinical study of 440 adult patients receiving cefepime-taniborbactam for treatment of cUTIs included headache, gastrointestinal events (diarrhea, constipation, nausea, and abdominal distension), and hypertension (6). The incidence of these TEAEs ranged from 2% and 6.1% and severity was mild to moderate. The overall safety profile of cefepime-taniborbactam was similar in clinical studies of healthy participants and patients with cUTIs (6, 12, 13).

In summary, a 4 h intravenous infusion of cefepime-taniborbactam (2 g cefepime and 0.5 g taniborbactam) every 8 h for six consecutive doses was safe and well tolerated in healthy adult participants. Cefepime and taniborbactam concentrations in ELF were lower than the total or unbound plasma concentrations. The DPR_ELF/plasma_ ranged from 0.197 to 0.373 for cefepime and 0.153 to 0.253 for taniborbactam and was dependent on which aspirate of recovered BAL fluid was used to calculate ELF volume. The results demonstrate the penetration of taniborbactam into the lung and support further considerations of cefepime-taniborbactam as a potential treatment for severe bacterial pneumonia caused by susceptible Gram-negative pathogens.

## MATERIALS AND METHODS

The study was conducted between June 2021 and September 2021 at Pulmonary Associates, PA, Phoenix, Arizona. The principal investigator conducted all aspects of this study in accordance with US FDA regulations, International Council for Harmonisation E6 (R2) Good Clinical Practice, and applicable local, state, and federal laws. Study protocol, protocol amendments, informed consent form, investigator’s brochure, and other relevant documents were submitted and approved by Advarra Institutional Review Board (Columbia, MD) before beginning the study. Written informed consent was obtained from each subject at the time of enrollment and before any study procedures were performed.

### Study design

This is a phase 1 single-center, multiple-dose, open-label study to assess the safety and intrapulmonary pharmacokinetics of cefepime and taniborbactam in healthy adult male and female participants. Each subject received 4 h intravenous infusions of 2 g cefepime in combination with 0.5 g taniborbactam every 8 h for six consecutive doses. Blood samples were collected from all participants to measure cefepime and taniborbactam concentrations in plasma at 0 h (pre-dose) and 1, 2, 3, 3.95, 4.05, 4.25, 4.5, 5, 6, and 8 h after the start of the sixth intravenous dose of cefepime-taniboractam. Blood samples were also collected to determine plasma concentrations of cefepime and taniborbactam before (pre-dose, within 15 min of dosing) the administration of the third, fourth, and fifth doses of cefepime/taniborbactam to confirm that steady-state in plasma has been reached.

Each subject had a single standardized bronchoscopy and BAL scheduled at 1, 3, 4.25, 5, 6, or 8 h after the start of the sixth intravenous infusion of cefepime-taniborbactam. The standardized bronchoscopy and BAL procedure has been previously described (26–30). Four 50 mL aliquots of sterile saline were instilled, and each instillation was immediately aspirated and placed on ice as separate samples (aspirate 1, 2, 3, and 4). The time of each 50 mL aliquot of sterile saline instillation, the time of the BAL sample collection, and the volume of each aspirate were recorded. A blood sample to determine the concentration of urea was obtained during the bronchoscopy procedure (approximating the time of collection for the second aspirate of BAL). Aliquots of BAL fluid were obtained to determine urea concentrations in each BAL sample.

### Study population

Male and female participants had to fulfill all of the following inclusion criteria to be eligible for participation in this study: (i) between 18 and 55 years of age; (ii) body mass index of ≥18 kg/m^2^ and ≤32 kg/m^2^; (iii) body weight of >50 kg; (iv) forced expiratory volume in 1 s (FEV_1_) of at least 80% of the predicted value at screening; (v) have suitable venous access for intravenous drug administration and blood sampling; (vi) non-smoker, non-vaper (defined as no use of tobacco, nicotine or marijuana-containing products in any form within 3 months before study screening visit; (vii) a negative drug test for cocaine, cannabinoids, amphetamines, barbiturates, benzodiazepines, methadone and opiates, alcohol, and cotinine test at the screening visit or the time of confinement (day −1); (viii) males who are not surgically sterilized and females of childbearing potential must agree to use highly effective methods of contraception (as defined per protocol) during the study until 90 days after the last dose of study drug; (ix) female participants on effective methods of birth control agree to not change method of contraception for 90 days after the last dose of study drug; (x) ability and willingness to abstain from alcohol, caffeine, xanthine-containing beverages or food, or products containing any of these 48 h prior to the time of confinement (day −1) until discharge from the clinical study site; and (xi) ability and willingness to not engage in strenuous physical activity, sunbathing, and contact sports 48 h prior to the time of confinement (day −1) until discharge from the clinical study site.

Participants were excluded from participation in this study if there was evidence of any of the following at screening or confinement, as appropriate: (i) history or presence of significant oncological, cardiovascular, pulmonary, hepatic, renal, hematological, gastrointestinal, endocrine, immunological, dermatological, vascular or neurological disease, including any acute illness or surgery within the past 3 months determined by the investigator to be clinically relevant; (ii) known or suspected *Clostridioides difficile* infection within the past 6 months; (iii) employee or family member of clinical study site, contract research organization or the sponsor; (iv) positive test result for HIV antibody, hepatitis B virus surface antigen, or hepatitis C virus antibody; (v) positive reverse transcriptase polymerase chain reaction testing for severe acute respiratory syndrome of coronavirus 2 (SARS-CoV-2) at screening and at the time of confinement; (vi) presence of the following symptoms within 14 days to screening, at screening or day −1: fever, difficulty breathing, cough, sore throat, new or recent loss of taste or smell, nausea, vomiting, or diarrhea; (vii) close contact with a person who has tested positive for SARS-CoV-2 infection within 14 days prior to screening or at the time of confinement; (viii) subject with previous COVID-19 with known or suspected pulmonary involvement within 2 months of screening; (ix) ECG with QTcF interval duration ≥450 ms for males and ≥470 ms for females at screening or the time of confinement; and (x) participants who had any of the following abnormalities on laboratory values at screening or time of confinement: white blood cell count of <3,000 cells/mm^3^, hemoglobin concentration of <11 g/dL, absolute neutrophil count of <1,200 neutrophils/mm^3^, platelet count of <120,000 platelets/mm^3^, and alanine aminotransferase and aspartate aminotransferase values greater than two times the upper limit of normal for the reference laboratory.

Participants were also excluded from participation if any of the following criteria were met: (i) a recent history of alcohol consumption exceeding two standard drinks per day on average (1 standard drink = 10 g of alcohol); (ii) use of prescription medications (with the exception of acetaminophen [doses ≤ 3 g/day up to commencement of study], continued use of hormonal contraceptives, and topical lidocaine for bronchoscopy procedure), over-the-counter medications, health supplements, and herbal remedies (e.g., St. John’s Wort extract) must have been stopped at least 7 days prior to confinement and throughout the entire study, unless agreed to be not clinically relevant by the principal investigator; (iii) history of any hypersensitivity reaction following administrations of a cephalosporin, penicillin, or other β-lactam antibacterial drug or any component of cefepime or taniborbactam; (iv) history of significant multiple and/or severe allergies (including latex allergy), anaphylactic reaction, or significant prescription drug, non-prescription drug, or food intolerance; (v) a known history of a clinically significant hypersensitivity reaction or anaphylaxis to any medication (including topical medications) used during the bronchoscopy; (vi) donation of blood or plasma within 30 days to time of confinement, or whole blood loss of more than 500 mL within 30 days prior to confinement, or receipt of a blood transfusion within 1 year of study enrollment; (vii) participation in another investigational clinical trial within 30 days prior to screening; (viii) female participants who are pregnant, lactating, or planning to become pregnant during this study or within 90 days after the last dose of study drug; and (ix) male subjects with a female partner who was pregnant or lactating during the study or planning to attempt to become pregnant during this study or within 90 days after the last dose of study drug.

### Drug and urea assays

Cefepime and taniborbactam concentrations in plasma and BAL fluid were measured using a validated method with liquid chromatography-tandem mass spectrometry (LC/MS/MS) analysis at PRA International (Lenexa, KS) (12, 31). Urea concentrations in plasma and BAL fluid were measured by a validated LC/MS/MS method at Keystone Bioanalytical, Inc. (North Wales, PA) (26). Inter- and intra-assay accuracy and precision were established using standardized validation procedures for each method and matrix.

### Pharmacokinetic calculations of plasma concentrations

Non-compartmental methods were used to generate pharmacokinetic parameters for cefepime and taniborbactam in plasma. Maximum plasma concentration (C_max_), time to C_max_ (T_max_), and minimum plasma concentration (C_min_) were read from the observed plasma concentration-time profile after the start of the sixth dose of cefepime/taniborbactam. The C_min_ was defined as the lowest plasma concentration during the 8 h dosing interval, and after the sixth dose was administered. AUC with the sixth dose was computed with the linear-log trapezoidal method and microcomputer program, Phoenix WinNonLin (version 8.3, Certara USA, Inc., Princeton, New Jersey). The AUC values included were from time 0 to the time of the last quantifiable concentration (AUC_0–last_) and from time 0 to 8 h (AUC_0–8_). The plasma clearance (CL) and apparent volume of distribution at steady-state (V_ss_) were determined. The elimination rate constant (K_el_) was determined by linear regression, and the elimination half-life (t_1/2_) was calculated with the equation t_1/2_ = 0.693/K_el_.

### Calculations of ELF volume and antibiotic concentrations in ELF

The estimations of the ELF volume and drug concentrations in ELF were calculated with each BAL fluid supernatant from individual aspirates recovered from the first, second, third, and fourth instillations. The concentration of cefepime and taniborbactam (ABX_ELF_) in the ELF was determined as follows:


$$ABX_{ELF}=ABX_{BAL}\;\times \;(V_{BAL}\;/\;V_{ELF}),$$

where ABX_BAL_ is the measured concentration of cefepime or taniborbactam in BAL fluid, V_BAL_ is the volume of aspirated BAL fluid, and V_ELF_ is the apparent volume of ELF sampled by the BAL. V_ELF_ is derived from the following:


$$V_{ELF}=V_{BAL}\;\times \;Urea_{BAL}\;/\;Urea_{P},$$

where Urea_BAL_ is the concentration of urea in BAL fluid and Urea_P_ is the concentration of urea in plasma (17).

Subject-level ELF concentrations derived from a BAL volume-weighted averaging from all except the first BAL aspirate sample (i.e., excluding BAL aspirate sample 1) were determined for each subject and bronchoscopy sampling time:


$$ABX_{ELF\text{-}2+3+4}=[(ABX_{ELF\text{-}2}\times V_{ELF\text{-}2})+(ABX_{ELF\text{-}3}\times V_{ELF\text{-}3})+(ABX_{ELF\text{-}4}\times V_{ELF\text{-}4})]\;/\;V_{ELF\text{-}2+3+4},$$

where ABX_ELF-2+3+4_ is the pooled ELF concentration of cefepime and taniborbactam from BAL aspirates 2, 3, and 4, ABX_ELF-#_ and V_ELF-#_ are the individual values for each BAL aspirate sample (i.e., # = 2, 3, and 4), and V_ELF-2+3+4_ is the total volume of ELF for BAL aspirate samples 2, 3, and 4.

A second subject-level ELF concentration derived from a BAL volume-weighted averaging from all BAL aspirate samples was determined for each subject and bronchoscopy sampling time:


$$ABX_{ELF\text{-}1+2+3+4}=[(ABX_{ELF\text{-}1}\times V_{ELF\text{-}1})+(ABX_{ELF\text{-}2}\times V_{ELF\text{-}2})+(ABX_{ELF\text{-}3}\times V_{ELF\text{-}3})+(ABX_{ELF\text{-}4}\times V_{ELF\text{-}4})]\;/\;V_{ELF\text{-}1+2+3+4},$$

where ABX_ELF-1+2+3+4_ is the pooled ELF concentration of cefepime and taniborbactam from all BAL aspirates (i.e., 1, 2, 3, and 4), ABX_ELF-#_ and V_ELF-#_ are the individual values for each BAL aspirate sample (i.e., # = 1, 2, 3, and 4), and V_ELF-1+2+3+4_ is the total volume of ELF for all BAL aspirate samples (i.e., 1, 2, 3, and 4).

The arithmetic mean concentration values of cefepime and taniborbactam at each BAL sampling time (e.g., 1, 3, 4.25, 5, 6, and 8 h) were calculated and used as a single data set to estimate a composite AUC_0–8_ of plasma and ELF. The elimination rate constant (K_el-ELF_) was determined by linear regression, and the elimination half-life (t_1/2-ELF_) for ELF was calculated with the equation, t_1/2-ELF_ = 0.693/ K_el-ELF_. The AUC_0–8_ for individual and pooled aspirates of each matrix was determined with the linear-log trapezoidal method using Phoenix WinNonLin software. The concentration at the 8 h sampling time also served as a value at time zero for determining the AUC_0–8_ of plasma and ELF. Drug penetration ratio into ELF (DPR_ELF/plasma_) was calculated by dividing the AUC_0–8_ value for ELF by the AUC_0–8_ value for unbound plasma. In addition, concentration ratios of ELF to the simultaneous unbound plasma concentration were calculated for each subject and summarized per group at each sampling time. Unbound fraction values used for cefepime and taniborbactam in plasma were based on previous studies and were 0.776 and 1.00, respectively (13).

### Safety assessment

Safety was assessed throughout the study by adverse event monitoring, clinical laboratory tests (hematology, biochemistry, and urinalysis), safety ECGs, physical examinations, body temperatures, and vital sign monitoring. Safety data were summarized, and the incidence of adverse events was presented by system organ class, the relationship to the study medication, and severity. Descriptive statistics were calculated for quantitative safety data, and frequency counts were used for the classification of qualitative safety data.

## SUPPLEMENTAL MATERIAL

Supplemental tables aac.00493-25-s0001.pdf Tables S1 to S5.

ASM does not own the copyrights to Supplemental Material that may be linked to, or accessed through, an article. The authors have granted ASM a non-exclusive, world-wide license to publish the Supplemental Material files. Please contact the corresponding author directly for reuse.
