# Supplementary material for: Plasma and intrapulmonary pharmacokinetics of cefepime and taniborbactam in healthy adult participants
Source: Antimicrob Agents Chemother. 2025 Jun 6;69(7):e00493-25. doi: 10.1128/aac.00493-25 (PMC12217482; doi:10.1128/aac.00493-25)
Supplement: Supplemental tables — Tables S1 to S5. [file aac.00493-25-s0001.pdf]

Supplementary Tables

Plasma and Intrapulmonary Pharmacokinetics of Cefepime and Taniborbactam in Healthy Adult Participants

Keith A. Rodvold, Pharm.D.[1#]

Mark H. Gotfried, M.D.[1,2]

Philip Sabato, Pharm.D., M.S.[3,4]

Tim Henkel, M.D., Ph.D.[3]

Paul C. McGovern, M.D.[3]

[1] University of Illinois Chicago, Chicago, Illinois, USA

[2] Pulmonary Associates, PA, Phoenix, Arizona, USA

[3] Venatorx Pharmaceuticals, Malvern, Pennsylvania USA

[4] Currently at Deciphera Pharmaceuticals, Waltham, Massachusetts USA

Short Title: Intrapulmonary PK of Cefepime and Taniborbactam

Table S1. Demographic characteristics[a] of 30 healthy nonsmoking adult participants receiving 4-hour intravenous infusion of 2 g cefepime in combination with 0.5 g taniborbactam every 8 hours for six doses

	Sex	Age (years)	Weight (kg)	Height (cm)	BMI (kg/m^2)	CL_{CR} (mL/min)
All Participants[b]	21 M, 9F	40 ± 8	81.8 ± 12.8	172 ± 10	27.5 ± 3.2	114 ± 20
1-hour Cohort[c]	4 M, 1F	42 ± 8	86.5 ± 7.4	175 ± 9	28.1 ± 2.4	115 ± 27
3-hour Cohort[c]	2 M, 3F	45 ± 12	70.4 ± 17.3	167 ± 7	24.8 ± 4.2	100 ± 17
4.25-hour Cohort[c]	4 M, 1F	40 ± 10	84.2 ± 8.1	175 ± 9	27.7 ± 1.4	103 ± 9
5-hour Cohort[c]	5 M	42 ± 7	91.3 ± 4.8	179 ± 6	28.6 ± 1.3	123 ± 27
6-hour Cohort[c]	5 M	38 ± 8	88.4 ± 10.1	177 ± 7	28.4 ± 3.3	131 ± 9
8-hour Cohort[c]	1 M, 4F	36 ± 3	70.2 ± 10.7	161 ± 11	27.2 ± 4.8	112 ± 8

[a] Data are expressed as arithmetic mean ± SD except for sex (presented as number of participants)
[b] 30 participants
[c] 5 participants per cohort
M = male; F = female
BMI = body mass index
CL_{CR} = Calculated creatinine clearance based on Cockcroft-Gault equation[11]

Table S2. Cefepime concentrations[a] in epithelial lining fluid (ELF) for BAL aspirates and at assigned BAL sampling times (N=5/timepoint)

BAL Sampling Time	Aspirate 1 (μg/mL)	Aspirate 2 (μg/mL)	Aspirate 3 (μg/mL)	Aspirate 4 (μg/mL)	Aspirates 2+3+4 (μg/mL)	Aspirates 1+2+3+4 (μg/mL)
1-hour	12.89 ± 5.69	9.28 ± 2.60	7.84 ± 3.07	9.17 ± 3.47	8.78 ± 3.01	9.14 ± 3.00
3-hour	19.83 ± 6.07	15.36 ± 3.25	12.75 ± 2.59	10.83 ± 1.84	12.56 ± 1.19	13.10 ± 0.96
4.25-hour	15.20 ± 6.18	11.39 ± 2.38	8.20 ± 1.41	7.45 ± 1.23	8.67 ± 1.42	9.70 ± 2.03
5-hour	10.04 ± 1.21	6.88 ± 1.61	6.30 ± 1.85	5.91 ± 1.58	6.34 ± 1.53	6.71 ± 1.42
6-hour	8.85 ± 1.28	5.96 ± 0.45	4.62 ± 0.81	3.49 ± 0.62	4.43 ± 0.68	4.79 ± 0.84
8-hour	6.29 ± 3.73	3.99 ± 1.64	2.86 ± 1.38	1.88 ± 0.68	2.83 ± 1.13	3.15 ± 1.31

[a] Data are expressed as arithmetic mean ± SD

Table S3. Taniborbactam concentrations[a] in epithelial lining fluid (ELF) for BAL aspirates and at assigned BAL sampling times (N=5/timepoint)

BAL Sampling Time	Aspirate 1 (μg/mL)	Aspirate 2 (μg/mL)	Aspirate 3 (μg/mL)	Aspirate 4 (μg/mL)	Aspirates 2+3+4 (μg/mL)	Aspirates 1+2+3+4 (μg/mL)
1-hour	3.02 ± 1.09	2.37 ± 0.66	2.31 ± 0.78	2.38 ± 0.84	2.36 ± 0.72	2.43 ± 0.71
3-hour	4.20 ± 0.62	3.12 ± 0.54	2.68 ± 0.19	2.63 ± 0.63	2.78 ± 0.45	2.88 ± 0.44
4.25-hour	3.45 ± 1.48	2.92 ± 0.60	2.18 ± 0.41	1.85 ± 0.42	2.22 ± 0.47	2.34 ± 0.59
5-hour	2.40 ± 0.84	1.86 ± 0.47	1.52 ± 0.52	1.36 ± 0.44	1.56 ± 0.45	1.63 ± 0.46
6-hour	1.98 ± 0.40	1.39 ± 0.09	1.15 ± 0.20	1.01 ± 0.14	1.14 ± 0.18	1.22 ± 0.22
8-hour	1.27 ± 0.46	0.87 ± 0.20	0.73 ± 0.13	0.57 ± 0.08	0.71 ± 0.11	0.76 ± 0.13

[a] Data are expressed as arithmetic mean ± SD

Table S4. Cefepime concentration ratio[a] in epithelial lining fluid (ELF) to unbound plasma for BAL aspirates and at assigned BAL sampling times (N=5/timepoint)

BAL Sampling Time	Aspirate 1	Aspirate 2	Aspirate 3	Aspirate 4	Aspirates 2+3+4	Aspirates 1+2+3+4
1-hour[a]	0.322 ± 0.068	0.239 ± 0.041	0.199 ± 0.048	0.235 ± 0.072	0.224 ± 0.052	0.234 ± 0.047
3-hour[a]	0.367 ± 0.110	0.282 ± 0.051	0.233 ± 0.027	0.202 ± 0.050	0.232 ± 0.028	0.242 ± 0.027
4.25-hour[a]	0.333 ± 0.105	0.256 ± 0.050	0.183 ± 0.018	0.166 ± 0.015	0.194 ± 0.012	0.207 ± 0.019
5-hour[a]	0.350 ± 0.040	0.238 ± 0.040	0.218 ± 0.052	0.205 ± 0.046	0.220 ± 0.038	0.233 ± 0.036
6-hour[a]	0.437 ± 0.075	0.295 ± 0.045	0.225 ± 0.024	0.170 ± 0.021	0.216 ± 0.008	0.233 ± 0.014
8-hour[a]	0.684 ± 0.404	0.433 ± 0.160	0.315 ± 0.152	0.210 ± 0.084	0.311 ± 0.122	0.345 ± 0.137

[a] Data are expressed as arithmetic mean ± SD

Table S5. Taniborbactam concentration ratio[a] in epithelial lining fluid (ELF) to unbound plasma for BAL aspirates and at assigned BAL sampling times (N=5/timepoint)

BAL Sampling Time	Aspirate 1	Aspirate 2	Aspirate 3	Aspirate 4	Aspirates 2+3+4	Aspirates 1+2+3+4
1-hour	0.234 ± 0.043	0.186 ± 0.026	0.182 ± 0.049	0.187 ± 0.049	0.185 ± 0.037	0.190 ± 0.033
3-hour	0.240 ± 0.039	0.180 ± 0.043	0.153 ± 0.026	0.151 ± 0.044	0.160 ± 0.035	0.166 ± 0.034
4.25-hour	0.240 ± 0.097	0.204 ± 0.035	0.152 ± 0.024	0.129 ± 0.025	0.155 ± 0.026	0.163 ± 0.034
5-hour	0.248 ± 0.054	0.192 ± 0.017	0.156 ± 0.028	0.140 ± 0.026	0.161 ± 0.020	0.169 ± 0.020
6-hour	0.282 ± 0.066	0.198 ± 0.033	0.161 ± 0.013	0.142 ± 0.012	0.160 ± 0.011	0.170 ± 0.017
8-hour	0.388 ± 0.155	0.261 ± 0.068	0.226 ± 0.075	0.175 ± 0.055	0.218 ± 0.062	0.234 ± 0.066

[a] Data are expressed as arithmetic mean ± SD
